# Comparison of Pelvic Floor Dysfunction in Women with Ulcerative Colitis and Healthy Population

**DOI:** 10.34172/mejdd.2024.384

**Published:** 2024-07-31

**Authors:** Maryam Soheilipour, Babak Tamizi Far, Razieh Fadaei, Peyman Adibi

**Affiliations:** ^1^Gastroenterology and Hepatology Research Center, Isfahan University of Medical Sciences, Isfahan, Iran

**Keywords:** Pelvic floor dysfunction, Inflammatory bowel disease, Ulcerative colitis, Urinary distress inventory, Colo-Rectal-Anal distress inventory

## Abstract

**Background:**

The possibility of pelvic floor dysfunction (PFD) occurrence seems to be higher in patients with inflammatory bowel disease (IBD) due to the presence of functional gastrointestinal disorders in these patients. Hence, this study aimed to evaluate the association of ulcerative colitis (UC) in women with PFD and its comparison with the healthy (without IBD) population.

**Methods:**

The present study was conducted on 150 women with UC and 150 without-IBD individuals. Pelvic Floor Distress Inventory (PFDI-20) was used to evaluate the pelvic floor function.

**Results:**

The results of this study revealed that UC had a significant role in increasing not only the PFD score (Beta=3.04; *P*<0.001) but also the score of each sub-scale of Pelvic Organ Prolapse Distress Inventory (POPDI) (Beta=6.61; *P*<0.001), Colo-Rectal-Anal Distress Inventory (CRADI) (Beta=9.37; *P*<0.001), and Urinary Distress Inventory (UDI) (Beta=5.56; *P*=0.015). In addition, aging, increased body mass index (BMI) and menopause had significant role in increasing POPDI, UDI, and PFDI scores, respectively (*P*<0.05).

**Conclusion:**

The percentage of PFD in women with UC was significantly higher than its percentage in women without IBD. This dysfunction was more visible in the two sub-scales of POPDI and CRADI. In addition to having UC, aging, BMI, and menopause played a significant role in increasing PFD.

## Introduction

 Inflammatory bowel diseases (IBDs) are chronic, relapsing, and remitting inflammatory diseases of the gastrointestinal tract. Although most patients recover after taking medicine.^1,2^ Many patients, despite the obvious remission of the disease caused by medication or surgery, still face some intestinal problems such as fecal urgency, increase in frequent bowel movement, fecal incontinence (FI), constipation (incomplete bowel movement), abdominal pain, or bloating.^3-5^

 In this regard, Pelvic floor dysfunction (PFD) is one of the most common problems for these patients. PDF comprises obstructed defecation, rectocele (colon prolapse), pelvic floor prolapse, functional constipation, dyssynergic defecation, paradoxical puborectalis contraction, coccyalgia, and pelvic floor spasm syndrome. FI is a major concern for patients with IBD^6,7^ such that its prevalence in these patients has been reported to be more than 24%, which mostly occurs in the active and silent stages of the disease.^8-12^ In addition, the prevalence of constipation has been reported in 26% and 6% of patients with ulcerative colitis (UC) and Crohn’s disease during recovery, respectively.^3^ Incomplete bowel movement problems have been reported in 9-40% of patients with ileo-anal pouch and increases with aging,^13-15^ Despite their high prevalence, these symptoms are less reported by patients and not diagnosed by physicians.^9,16,17^ Most likely, a complex interaction of physiological and psychological factors is involved in the development and continuation of functional gastrointestinal symptoms after recovery from the disease.^3,18-20^

 Changes in bowel movement, sensitivity, and contraction occurring in response to the inflammatory process, as well as rectal or pouch compliance (stiffness), play a role in causing symptoms,^21-23^ Psychological stress affects bowel movements, visceral sensation, and immune factors and can exacerbate or perpetuate symptoms. Persistent symptoms can be associated with anxiety, depression, absenteeism, use of health care services, and impaired quality of life.^24-26^

 PFD may be in response to unpleasant stimuli such as abdominal or rectal pain, loose stools, and fecal urgency, which are common in patients with IBD.^27^ When pelvic floor and anal sphincter muscles contract or are not loose enough during defecation, bowel movements become obstructed. This process is recognized as dyssynergia, paradoxical puborectalis contraction, or non-relaxing pelvic floor muscle dysfunction.^28,29^

 PFD has been detected in more than 50% and 45%-97% of patients with ileo-anal pouch and silent or controlled IBD, respectively.^30,31^

 In addition, one of the groups prone to facing these disorders is women. Urinary and fecal incontinence is seen in 17%-45% and 4%-17% of adult women, respectively, and their prevalence increases with aging.^32,33^ Several epidemiological studies in different populations consider several factors involved in PFD. Furthermore, uterine-vaginal laxity is a common problem and constitutes about 20% of women’s surgeries in developed countries.^34^ Predisposing factors of these disorders include aging, delivery, obesity, and menopause.^35^ Of course, the role of injuries caused by vaginal delivery as a contributing factor for PFD is well acknowledged.^36^

 According to the presented literature, it seems that PFD is prevalent in patients with IBD and has been mentioned in many previous studies. However, as the occurrence of this condition in women with silent IBD has not been evaluated in any study, the present study enjoyed acceptable innovation. Moreover, it should be noted that this population should receive due attention as women can be one of the high-risk groups prone to this disorder, and if suffering from silent IBD, they will have a greater chance of facing PFD. Hence, considering the prevalence and effect of functional gastrointestinal symptoms in patients with silent IBD, as well as the great importance of these problems, it is indispensable to recognize the potential predisposing factors in the development of these disorders to prevent or at least reduce them. Therefore, the present study aimed to evaluate the association of UC in women with PFD and compare it with the (healthy) without-IBD population.

## Materials and Methods

 The current study was cross-sectional. The study population included all women with IBD, whose information was recorded in the IBD registry system of Al-Zahra hospital during 2020-2022.

 It should be taken into account that due to the significance of this study and the limited sample eligible to enter the study, all eligible women with controlled IBD (only UC), approximately 150 individuals, were included in the study as the patient group. Moreover, each of these patients’ friends, that were 150 individuals without symptoms of IBD (as a healthy group without IBD) were requested to participate in the study.

 The criteria for entering the study in the patient group were women with UC, a controlled state (remission) of the disease (no evidence of disease activity in colonoscopy in the last month or calprotectin less than 50 μg/mg), the age range of 18-60 years, the necessary information documented in the IBD registry system to contact the patient, and written consent to participate in the study. The patients were not included in the study in case of patients’ non-response to the phone, non-communication with the patients, or patient death. Moreover, the without-IBD individuals were not involved in the study in case of any evidence regarding their gastrointestinal disorders, previous records of IBD, and previous referrals to a gastroenterologist.

 After obtaining the code of ethics from the Ethics Committee of Isfahan University of Medical Sciences (approval code: IR.ARI.MUI.REC.1401.108), the contact number of women with controlled IBD (only UC) was extracted from the IBD registry system of Al-Zahra hospital. They were contacted and requested to participate in the study after explaining the purpose of the study. They were also asked to introduce one of their friends who did not suffer from IBD or any other gastrointestinal disorders for the control group.

 Then, the eligible individuals’ demographic and clinical characteristics consisting of their age, height, weight, body mass index (BMI), education status, marital status, employment status, number of deliveries, type of delivery, comorbidities (such as hypertension, kidney, cardiovascular, pulmonary problems, etc.), menopause, smoking, hookah consumption, addiction, type of UC (pancolitis, left colitis or limited, proctosigmoiditis, proctitis), medicines used (biological medicines, cytotoxic medicines, etc.) were recorded.

 Moreover, the Pelvic Floor Distress Inventory (PFDI-20) was filled out for them over the phone.

 This questionnaire was designed by Barber and colleagues in 2005 and included 20 items measuring pelvic disorders in three sub-scales of prolapse symptoms (POPDR-6), anorectal symptoms (CRADI-8), and urinary symptoms (UDI-6).^36^ Each item was scored from 0 to 4. If the answer was no, a score of zero was assigned. Moreover, if the answer was yes, a score of 1 to 4 (according to the severity of the symptoms) was allocated. The scores of each sub-scale were calculated within the range of 0-100, and the total score was considered within the range of 0-300 by adding the scores of three sub-scales. A higher score indicated a greater effect of PFD on the individual’s life. The validity and reliability of this questionnaire were examined in the thesis of Hakimi et al. According to this study, the validity of this questionnaire was 0.698, and its reliability was reported to be 0.723 based on Cronbach’s alpha coefficient.^37^

###  Data Analysis

 All information collected entered SPSS software version 26. At the level of descriptive statistics, indices such as mean, standard deviation, frequency, and frequency percentage were used. At the level of inferential statistics, the chi-square test, independent samples *t* test, and simple regression were used. The significance level was considered less than 0.05 in all analyses.

## Results

 In this study, out of 150 women with UC, 38.3%, 9%, 3.8%, and 48.1% had pancolitis, limited or left-sided colitis, proctosigmoiditis, and proctitis, respectively. The mean age of the UC and healthy groups was 44.73 ± 13.43 and 43.55 ± 11.49 years, respectively (*P* > 0.05). The weight and BMI of the UC group were significantly lower than the healthy group (*P* < 0.05). In addition, liver and rheumatoid diseases in the UC group, with percentages of 9.3% and 14.7% were significantly higher than these diseases in the healthy group with percentages of 3.3% and 4.7%, respectively (*P* < 0.05). However, patients’ other basic and clinical characteristics were not significantly different between the two groups (*P* > 0.05) (Table 1).

**Table 1:** Basic and clinical characteristics of the two studied groups

**Variables**		**Healthy group (n=150)**	**UC group (n=150)**	***P*** ** value**
Age (year)		43.55 ± 11.49	44.73 ± 13.43	0.417
Height (cm)		162.22 ± 6.75	163.02 ± 5.51	0.263
Weight (kg)		69.16 ± 12.49	65.76 ± 10.43	0.011
BMI (kg/m^2^)		28.03 ± 5.12	26.23 ± 3.12	0.047
Education status	Illiterate	4 (2.7%)	3 (2%)	0.716
	≤ Diploma	81 (54.0%)	82 (54.7%)	
	College education	65 (43.3%)	65 (43.3%)	
Employment status	Employed	50 (33.3%)	29 (19.3%)	0.056
	Housewife	100 (66.7%)	121 (80.7%)	
Marital status	Single	10 (6.7%)	12 (8.0%)	0.658
	Married	140 (93.3%)	138 (92.0%)	
Number of children		2.23 ± 1.69	2.18 ± 1.69	0.784
Without children		20 (13.3%)	14 (9.3%)	0.136
1-2 children		55 (36.7%)	78 (52%)	
> 2 children		75 (50%)	58 (38.7%)	
Type of delivery	None	20 (13.3%)	10 (6.7%)	0.306
	Vaginal delivery	51 (36.2%)	52 (37.7%)	
	Cesarean section	56 (39.7%)	62 (44.9%)	
	Both	14 (9.9%)	14 (10.1%)	
Comorbidities	Cardiovascular	5 (3.3%)	7 (4.7%)	0.770
	Kidney	5 (3.3%)	11 (7.3%)	0.198
	HTN	12 (8%)	21 (14.0%)	0.139
	DM	7 (4.7%)	11 (7.3%)	0.467
	Pulmonary disease	5 (3.3%)	9 (6.0%)	0.413
	Skin disease	6 (4.0%)	5 (3.3%)	0.989
	Cancer disease	0 (0.0%)	0 (0.0%)	-
	Gynecological disease	9 (6.0%)	18 (12.0%)	0.105
	Liver disease	5 (3.3%)	14 (9.3%)	0.035
	Rheumatic disease	7 (4.7%)	22 (14.7%)	0.005
Menopause		42 (28%)	46 (30.7%)	0.704
Smoking		5 (3.3%)	7 (4.7%)	0.770
Hookah consumption		3 (2%)	5 (3.3%)	0.802
Addiction		0 (0.0%)	0 (0.0%)	-
Type of UC	Pancolitis	-	51 (38.3%)	-
	Left colitis or Limited	-	12 (9.0%)	-
	Proctosigmoiditis	-	5 (3.8%)	-
	Proctitis	-	64 (48.1%)	-
Medicines used	5-ASA	-	143 (95.3%)	-
	Cytotoxic drugs	-	60 (40.0%)	-
	Biological drugs	-	21 (14.0%)	-

5-ASA: Aminosalicylic acid including mesalazine, ASACOL; Cytotoxic drugs including azathioprine, tacrolimus, mycophenolic acid; Biological drugs including remicade, adalimumab, BMI: body mass index

 Examination of PFD revealed that generally, the PFI score in UC women with a mean of 64.44 ± 41.33 was significantly higher than that of healthy women with a mean of 37.40 ± 39.72 (*P* > 0.001). The two sub-scales of POPDI and CARDI in UC women with the means of 18.32 ± 14.35 and 27.77 ± 18.19 were significantly higher than those of healthy women with the means of 12.83 ± 14.71 and 9.47 ± 13.83, respectively (*P* ≤ 0.001). However, there was no significant difference in the mean of UDI between the two studied groups (*P* > 0.05) (Table 2). It should be noted that 31.3% of healthy women and 1.3% of UC women had no PFD (with a score of zero) (*P* < 0.001), which can indicate a significant difference in the possible occurrence of PFD in UC women, as compared to healthy women.

**Table 2:** Comparison of the mean of PFDI between two studied groups

**PFDI**	**Healthy group (n=150)**	**UC group (n=150)**	***P*** ** value**
POPDI	12.83 ± 14.71	18.32 ± 14.35	0.001
CARDI	9.47 ± 13.83	27.77 ± 18.19	< 0.001
UDI	15.11 ± 18.00	18.34 ± 19.45	0.136
Total Score of PFDI	37.40 ± 39.72	64.44 ± 41.33	< 0.001

CRADI: Colo-Recto-Anal Distress Inventory; POPDI: Pelvic Organ Prolapse Distress Inventory; UDI: Urinary Distress Inventory, PFDI: Pelvic Floor Distress Inventory

 Moreover, IBD (only UC) had a significant role in increasing the total score of PFD (Beta = 3.04; *P* < 0.001) and increasing each sub-scale of POPDI (Beta = 6.61; *P* < 0.001), CARDI (Beta = 9.37; *P* < 0.001), and UDI (Beta = 5.56; *P* = 0.015). Besides, aging had a significant role in increasing the POPDI score (Beta = 0.14; *P* = 0.044). Furthermore, increasing BMI had a significant role in increasing the UDI score (Beta = 0.57; *P* = 0.026) while the menopause factor played a significant role in increasing the overall PFDI score (Beta = 4.39;* P* = 0.028) and UDI score (Beta = 5.55; *P* = 0.024). In contrast, the increase in education status had a significant role in reducing the total score of PFDI (Beta = -4.894; *P* = 0.045) (Table 3).

**Table 3:** Investigation of factors affecting PFD score

**Factors**	**Total Score of PFDI**			**POPDI**			**CARDI**			**UDI**		
	**Beta**	**SE**	***P*** ** value**	**Beta**	**SE**	***P*** ** value**	**Beta**	**SE**	***P*** ** value**	**Beta**	**SE**	***P*** ** value**
Group (UC vs. Healthy)	3.04	1.85	< 0.001	6.61	1.70	< 0.001	9.37	1.88	< 0.001	5.56	2.23	0.015
Age	0.09	0.28	0.732	0.14	0.07	0.044	-0.08	0.11	0.492	0.09	0.10	0.356
Education status	-4.89	2.14	0.045	-2.54	1.97	0.200	-1.15	2.18	0.598	-3.23	2.18	0.139
BMI	0.78	0.55	0.156	0.06	0.21	0.782	0.13	0.23	0.578	0.57	0.25	0.026
Marital status	3.06	3.96	0.395	1.67	4.29	0.456	1.08	1.72	0.481	1.72	1.62	0.495
Employment status	-1.46	1.23	0.095	-4.15	2.28	0.070	-3.55	2.20	0.108	-2.63	2.91	0.368
Number of children	1.93	1.89	0.309	-0.05	0.91	0.953	0.64	0.67	0.338	0.31	1.19	0.798
Type of delivery (vaginal vs. Cesarean section)	-0.24	3.22	0.941	0.72	1.14	0.577	-1.25	1.22	0.307	0.68	1.42	0.633
Menopause	4.39	1.15	0.028	1.11	2.77	0.689	3.39	3.04	0.266	5.55	2.44	0.024

PFDI: Pelvic Floor Distress Inventory; POPDI: Pelvic Organ Prolapse Distress Inventory, CRADI: Colo-Rectal-Anal Distress Inventory; UDI: Urinary Distress Inventory; BMI: body mass index

## Discussion

 According to the results of the present study, 31.3% of healthy women and 1.3% of UC women did not have PFD. Moreover, the scores of PFI and its two sub-scales (POPDI and CARDI) were significantly higher in UC women than in healthy women. However, the mean of UDI was not significantly different between the two groups.

 In line with the present study, Singh and colleagues investigated the association between PFD and chronic constipation and irritable bowel syndrome with constipation (IBS-C) and revealed that IBS-C and higher severity of constipation had a significant association with PFDI-20 scores, while dyssynergia had no significant association with PFDI-20 score. In addition, they showed that patients with IBS-C, as compared with patients with functional constipation, had significantly more discomfort from specific symptoms of the pelvic floor, including pelvic organ prolapse and lower urinary tract symptoms.^38^

 Furthermore, Bondurri and others’ study examining PFD in IBD patients showed that IBD patients faced a wide range of disorders, including urinary incontinence, fecal impaction, and pelvic pain. A special issue among fecal symptoms in patients with IBD is paradoxical puborectalis contraction after restorative proctocolectomy. In this case, it is suggested that a conservative treatment be chosen and unnecessary laparotomy avoided as much as possible.^39^ Although the main focus of the above-mentioned study was selecting an appropriate treatment for IBD patients with PFD, the significant point in this study was the association between IBD and the occurrence of PFD. In this respect, the findings of this study confirmed the results of our study.

 The results of Chou and colleagues’ study also indicated that patients with severe obstructed defecation symptoms had a lower PFDI-20 score than patients with severe colonic inertia symptoms.^40^ In addition, Norton and co-workers also stated that FI was seen in a higher percentage of patients with IBD.^9^

 Examining the role of IBD (UC women) along with patients’ other demographic and clinical factors in the PFD revealed that having UC had a significant role in increasing the score of not only PFD but also each of the sub-scales of POPDI, CARDI, and UDI. Moreover, aging had a significant role in increasing the POPDI score, while an increased BMI had a significant role in increasing the UDI score. Moreover, menopause played a significant role in increasing the overall PFDI score and UDI score. Hence, it can be stated that in addition to UC, age, BMI, and menopause can also be associated with an increased PFD.

 It should be considered that Letouzey and colleagues investigated whether PFDI could be used to prognosticate the outcome of pelvic reconstructive surgery or not and showed that PFDI could have a good diagnostic value on the success of pelvic reconstructive surgery and improvement of pelvic disorders. In this respect, there was a significant improvement 6 months after surgery in patients with PFDI that had a score of more than 62 before surgery; however, no improvement was achieved in patients with PFDI score of less than 62. In fact, they specified a cut-off point of 62 for this questionnaire and recommended that if the PFDI score is less than 62, medical treatments (hormonal or non-hormonal treatments) should be prescribed.^41^

 The results of Norton and colleagues’ study also indicated that age, sex, anal stretch, anal fistula surgery, colorectal surgery, and urinary incontinence had a significant association with the occurrence of FI; however, vaginal delivery had no significant association with the occurrence of this disorder.^9^ Although only FI disorder was investigated in the mentioned study, our study addressed PFD. Hence, these two studies are different in this regard. However, findings regarding the association of IBD with one of the pelvic disorders can be considered consistent with the findings of our study.

 In some other studies, it has been interpreted that the increase in FI in patients with IBD can probably be due to predisposing factors such as perianal disease, invasive perianal surgical approach, liquid stools, and secretory diarrhea caused by bile acid malabsorption.^42-44^

 Beer-Gabel and Carter performed a case-control study and figured out that patients with FC and IBS-C had no significant difference in terms of the frequency of lower urinary tract symptoms. Potentially IBS patients’ underlying higher visceral hypersensitivity involved the gut and other organ systems, which resulted in higher distress from non-gastrointestinal symptoms. Irritability of the bowel and bladder happens in IBS and overactive bladder (defined by urge incontinence and urinary urgency), respectively. Furthermore, both conditions were observed in many patients.^45^

 Singh and colleagues figured out higher distress from pelvic organ prolapse by POPDI-6 in patients with IBS-C. The mentioned finding can be largely attributed to lower abdominal pain, pressure, and heaviness during stool pass. Numerous studies have reported that IBS-C patients, as compared with FC patients, had higher overall abdominal pain. Actually, abdominal pain is considered an IBS-C diagnostic criterion. Possibly, the gastrointestinal tract and other pelvic floor-associated symptoms, including interstitial cystitis and pelvic organ prolapse, lead to higher abdominal pain in IBS-C patients.^38^

 Although several studies have investigated the association of individual pelvic floor complaints, such as pelvic organ prolapse, urinary symptoms, colorectal symptoms, etc, with chronic constipation, our study is one of the few studies that has investigated the association of IBD (only UC) with PFD using an assessment tool and can be valuable from this point of view. As another strong point, the female population has been of interest in this study, and due to the higher prevalence of pelvic, urinary, and bladder disorders among this population, paying attention to women with IBD problems can be of particular importance in the occurrence of PFD. In contrast, the small sample size and evaluation of only UC, failure to complete the questionnaire related to IBD symptoms in determining the severity of the disease and its duration, and lack of physical examination (use of questionnaire) can be considered as the drawbacks of this study. Hence, it is recommended that similar studies be conducted to obtain further and more generalizable findings in this respect.

## Conclusion

 According to the results of the present study, the PFD scores based on PFDI-20 and two sub-scales of POPDI and CARDI were significantly higher in UC women than in healthy (without IBD) women.
